# A Leader Intron of a Soybean *Elongation Factor 1A* (*eEF1A*) Gene Interacts with Proximal Promoter Elements to Regulate Gene Expression in Synthetic Promoters

**DOI:** 10.1371/journal.pone.0166074

**Published:** 2016-11-02

**Authors:** Ning Zhang, Leah K. McHale, John J. Finer

**Affiliations:** 1 Department of Horticulture and Crop Science, The Ohio State University, Wooster, Ohio, United States of America; 2 Department of Horticulture and Crop Science, The Ohio State University, Columbus, Ohio, United States of America; McGill University, CANADA

## Abstract

Introns, especially the first intron in the 5’ untranslated region (5’UTR), can significantly impact gene expression via intron-mediated enhancement (IME). In this study, we demonstrate the leader intron of a soybean *elongation factor 1A* (*eEF1A*) gene (GmScreamM8) was essential for the high activity of the native promoter. Furthermore, the interaction of the GmScreamM8 leader intron with regulatory element sequences from several soybean *eEF1A* promoters was studied using synthetic promoters, which consisted of element tetramers upstream of a core promoter used to regulate a *green fluorescent protein* (*gfp*) reporter gene. Element tetramers, placed upstream of a GmScreamM8 core promoter, showed very high activity using both transient expression in lima bean cotyledons and stable expression in soybean hairy roots, only if the native leader intron was included, suggesting an interaction between intronic sequences and promoter elements. Partial deletions of the leader intron showed that a 222 bp intronic sequence significantly contributed to very high levels of GFP expression. Generation of synthetic intron variants with a monomeric or trimeric repeat of the 222 bp intronic sequence, yielded almost two-fold higher expression compared to the original intron, while partial deletion of the 222 bp intronic repeated sequence significantly decreased gene expression, indicating that this intronic sequence was essential for the intron-element interaction enhancement.

## Introduction

Eukaryotic *elongation factor 1A* (*eEF1A*) genes, encoding one of the most abundant soluble proteins in plant cells [1], are constitutively expressed in most tissues, with high expression in rapidly growing tissues, such as shoot and root meristems and developing gametophytes [2]. Eukaryotic eEF1A proteins are active during the elongation phase of protein synthesis, catalyzing the binding of aminoacyl-tRNA to the acceptor site on the ribosome by a GTP-dependent pathway [3]. In addition, eEF1A proteins are multifunctional proteins and interact with major cytoskeletal proteins to organize the cytoskeleton structure by binding to tubulin, actin or other microtubule associated proteins [4]. In plants, eEF1As enhance tolerance to various forms of abiotic stress including wounding, low-temperature, high salt, and drought, possibly by elevating translational efficiency [5–8].

More than 80% of plant genes contain one or more introns, with an average density of four introns per gene [9]. Eukaryotic *eEF1A* genes exhibit a highly conserved structure, each with a relatively large intron in its 5’ untranslated region (5’ UTR). An Arabidopsis *eEF1A* promoter and its 5’ UTR intron was used in the herbicide resistance gene in commercial RoundupReady^®^ soybean plants [10], but the specific reason for use of those sequences has not been reported.

Since the discovery of the first introns in the 1970s [11], considerable effort has been placed into understanding the contributions of introns to gene regulation. Introns can either enhance or repress gene expression, depending on the DNA structure and the nucleotide content of intron flanking sequences [12]. Some introns can also regulate gene expression in a tissue- or development-specific manner [13–15], possibly by providing binding sites for specific transcription factors (TFs) *in vivo* under certain developmental or environmental conditions.

Introns, especially the first intron in the 5’UTR or the coding region (CDS) of a gene, can significantly enhance gene expression in both monocots and dicots [16–17], even though the magnitude of intron enhancement is usually much larger in monocots than in dicots [18]. Introns can enhance transcriptional efficiency either through an interaction between splicing factors and transcription factors, or by changing the chromatin structure into a transcriptionally active state by recruiting unique chromatin-remodeling proteins [19–22]. In addition, splicing signals can influence various stages of mRNA processing, including 5’ capping, 3’ polyadenylation, mRNA nuclear export, translation, and decay of mRNA products [19, 23–24]. At the DNA or RNA level, the presence of an intron affects the transcriptional/translational machinery [23, 25].

Introns seem to contain enhancers or regulatory elements that provide binding sites for transcription factors or activators [9]. For instance, a 5’ UTR intron in a soybean *polyubiquitin* (Gmubi) promoter augmented gene expression with properties of an enhancer, as placement of the intron upstream of the promoter region led to increased expression regardless of orientation [26]. In some cases, the intron itself can function as a promoter, driving weak but consistent gene expression [27–30]. However, in most cases, specific regulatory elements or enhancers contributing to gene expression enhancement cannot be identified from the intron sequence and intron enhancement of gene expression occurs only when the intron is placed within the transcribed region in the proper orientation, so that the intron can be appropriately processed. This type of intron enhancement, specifically termed “intron-mediated enhancement” (IME) [31], likely contributes to gene regulation via intron processing, even though the precise mechanism for enhancement is not completely understood.

Although IME has been widely studied, only a few studies have successfully identified specific intronic regulatory elements contributing to intron-mediated enhancement in plants [22, 25, 32–33]. We previously reported that three soybean (*Glycine max* Merr.) *eEF1A* promoters, each with a native 5’UTR intron, regulated high levels of gene expression [34]. In the present study, we investigated the effects of the 5’UTR intron from a soybean *eEF1A* gene (GmScreamM8) [34] on gene regulation by specifically studying the interaction of intronic sequences with a regulatory element from another highly-expressed soybean e*EF1A* gene (GmScreamM1) [34]. Synthetic promoters/introns consisting of an element tetramer, a core promoter, and variant intronic sequences were transcriptionally fused to a *green fluorescent protein* (*gfp*) reporter gene and evaluated using both transient expression in lima bean cotyledons and stable expression in soybean hairy roots. Use of the GmScreamM8 leader intron with proximal or core promoter regulatory elements led to enhanced gene expression.

## Materials and Methods

### Plasmid Constructs

**Fig 1:**
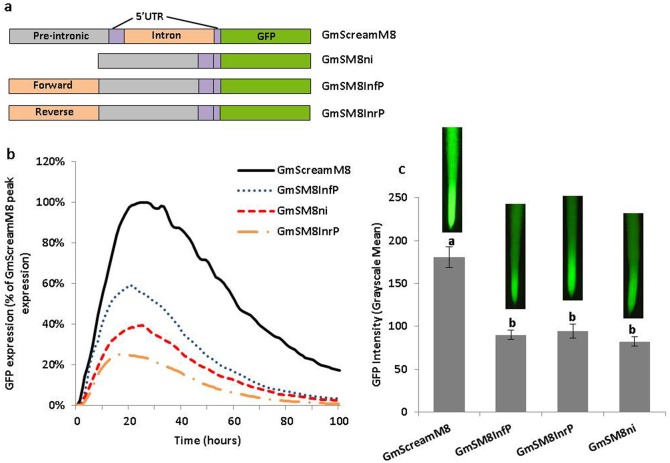
Validation of the leader intron on high activity of the GmScreamM8 promoter. (a) Schematic of intron deletion and translocation constructs. (b) Transient expression profiles for intron deletion and translocation constructs in lima bean cotyledons. The order of constructs in the figure caption key reflects the highest to lowest activity in the curves. (c) GFP intensity value (±SEM) in soybean hairy roots transformed with intron deletion and translocation constructs. Images of root tips on top of columns represent the average intensity of GFP expression driven by the corresponding promoter construct. Overlapping columns show no significant differences between constructs by the t test (LSD) at p<0.05.

**Fig 2:**
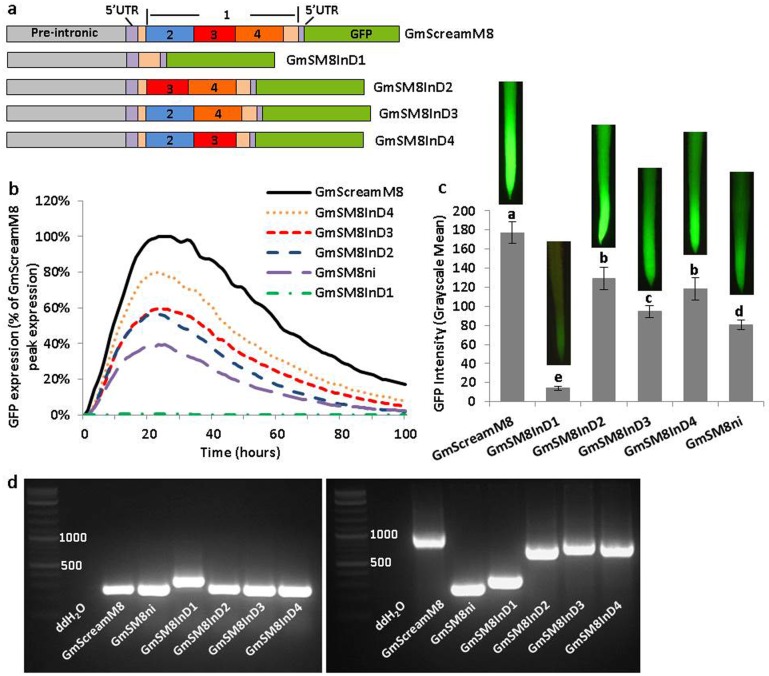
Partial deletion analysis of the leader intron in the GmScreamM8 promoter. (a) Schematic of intron partial deletion constructs in the GmScreamM8 promoter. (b) Transient expression profiles for intron partial deletion constructs in lima bean cotyledons. The order of constructs in the figure caption key reflects the highest to lowest activity in the curves. (c) GFP intensity value (±SEM) in soybean hairy roots regulated by different intron partial deletion constructs. Images of root tips on top of columns represent the average intensity of GFP expression driven by the corresponding promoter construct. Overlapping columns show no significant differences between constructs by the t test (LSD) at p<0.05. (d) Left: RT-PCR. Right: conventional PCR using plasmid DNA of each construct as template.

We previously reported the isolation of the GmScreamM8 promoter using PCR with soybean (*G*. *max* cv ‘Jack’) genomic DNA as template [34]. In this study, the GmScreamM8-pFLEV plasmid (GenBank accession number KX252734) was first used as a template to generate a series of GmScreamM8-derived intron variants with the full-length promoter (GmSM8ni, GmSM8InfP, GmSM8InrP, GmSM8InD series, Figs 1 and 2). Primers were designed to contain appropriate restriction sites at the 5’ end of each primer preceded by four or six additional nucleotides (S1 Table). To produce an intron-less version of the promoter (GmSM8ni-pFLEV [ni = no intron]), a reverse primer was specially designed to connect the first and second parts of the GmScreamM8 5’UTR that was originally interrupted by the native intron, while the forward primer was designed at the 5’ end of the GmScreamM8 promoter. To generate GmSM8InfP-pFLEV (Intron upstream of the pre-intronic promoter sequence in a forward orientation) and GmSM8InrP-pFLEV (Intron upstream of the pre-intronic promoter sequence in a reverse orientation) constructs, the 770 bp intronic region was first PCR-amplified using the GmSM8Inf(r)P_F and GmSM8Inf(r)P_R primer pair (S1 Table), in which a *Hin*dIII restriction site was introduced at both ends of the fragment. The amplified fragment was digested with *Hin*dIII and cloned into the corresponding site in the GmScreamM8-intronless construct (GmSM8ni-pFLEV) in both sense and antisense orientations. The orientation was confirmed by sequencing (Molecular and Cellular Imaging Center, OARDC, The Ohio State University, Wooster, Ohio, USA).

For a series of intron deletion variants, to make sure the intron was efficiently spliced, the 5’ splice site, 3’ splice site, and the branch site were retained in all intron variant constructs, while the rest of intronic sequence named “part 1” (= 702 bp) was further divided into three regions with a similar size, named “part 2” (= 269 bp), “part 3” (= 222 bp), and “part 4” (= 211 bp) (Fig 2a and S1 File). Four intron deletion variants (“GmSM8InD1”, “GmSM8InD2”, “GmSM8InD3”, and “GmSM8InD4”; InD = Intron Deletion) were generated using inverse PCR [35] with some modifications. Each pair of primers was designed in inverted tail-to-tail directions to amplify the whole plasmid sequence except for the targeted region to be deleted (S1 Table). PCR products were purified using a DNA Clean & Concentrator^‡^-5 kit (Zymo Research, Irvine, CA, USA), followed by 3’ blunting with DNA Polymerase I, large fragment (Klenow) and 5’ phosphorylation by T4 polynucleotide kinase. The PCR product was then incubated with 20 U *Dpn*I at 37°C for 1 h to remove the parental non-mutated plasmid DNA and then self-ligated using a Quick ligation kit (NEB, Ipswich, MA, USA) according to the manufacturer’s instructions. PCR-positive single colonies were identified and plasmid DNA was isolated and sequenced to confirm the sequence was correct.

**Fig 3:**
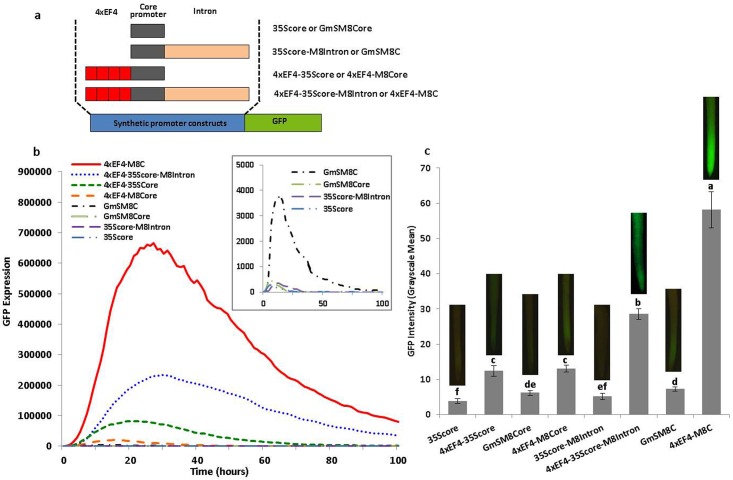
Validation of synthetic promoters containing the tetrameric element, the core promoter, and the leader intron. (a) Schematic of synthetic promoter constructs. (b) Transient expression profiles for different synthetic promoter constructs in lima bean cotyledons. The order of constructs in the figure caption key reflects the highest to lowest activity in the curves. GFP expression is presented as “GFP Expression” rather than percent of a control because expression values for two different core promoters are presented. (c) GFP intensity value (±SEM) in soybean hairy roots transformed with different synthetic promoter constructs. Images of root tips on top of columns represent the average intensity of GFP expression driven by the corresponding promoter construct. Overlapping columns show no significant differences between constructs by the t test (LSD) at p<0.05.

**Fig 4:**
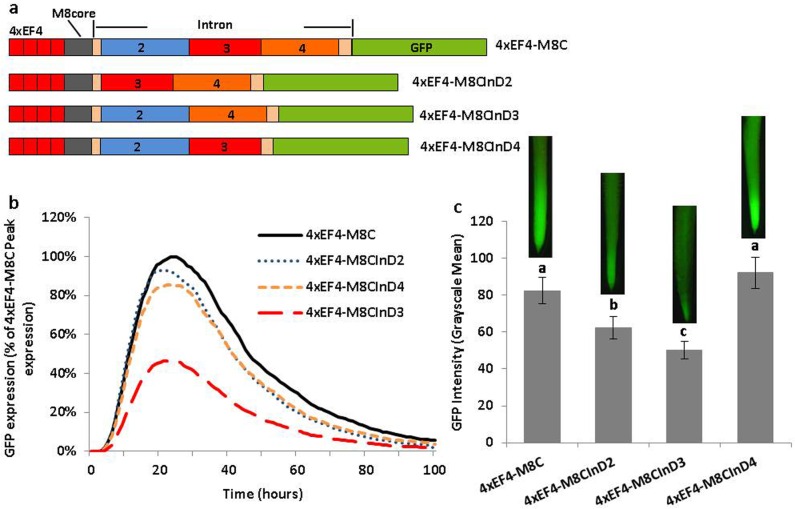
Partial deletion analysis of the leader intron using synthetic promoters. (a) Schematic of intron partial deletion constructs in synthetic promoters. (b) Transient expression profiles for intron deletion constructs in synthetic promoters in lima bean cotyledons. The order of constructs in the figure caption key reflects the highest to lowest activity in the curves. (c) GFP intensity value (±SEM) in soybean hairy roots transformed with intron deletion variants in synthetic promoters. Images of root tips on top of columns represent the average intensity of GFP expression driven by the corresponding promoter construct. Overlapping columns show no significant differences between constructs by the t test (LSD) at p<0.05.

**Fig 5:**
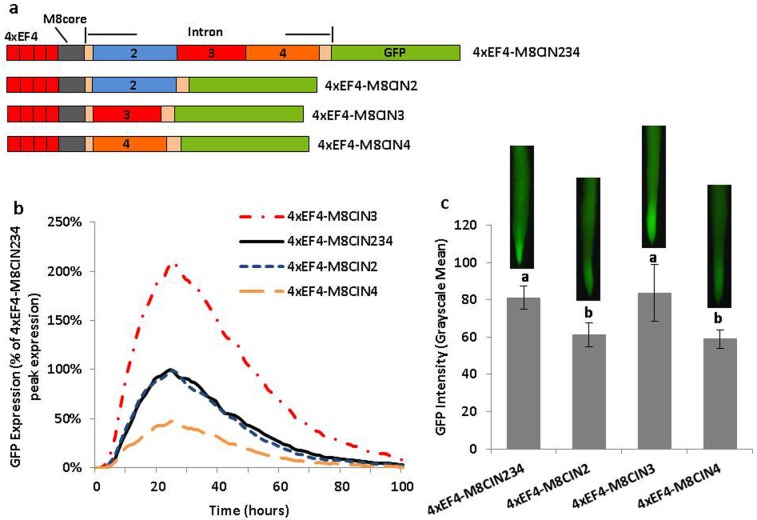
Cluster deletion analysis of the leader intron in synthetic promoters. (a) Schematic of all intron cluster deletion constructs. (b) Transient expression for different intron cluster deletion variants in lima bean cotyledons. The order of constructs in the figure caption key reflects the highest to lowest activity in the curves. (c) GFP intensity value (±SEM) in soybean hairy roots transformed with different intron cluster deletion variants in synthetic promoters. Images of root tips on top of columns represent the average intensity of GFP expression driven by the corresponding promoter construct. Overlapping columns show no significant differences between constructs by the t test (LSD) at p<0.05.

**Fig 6:**
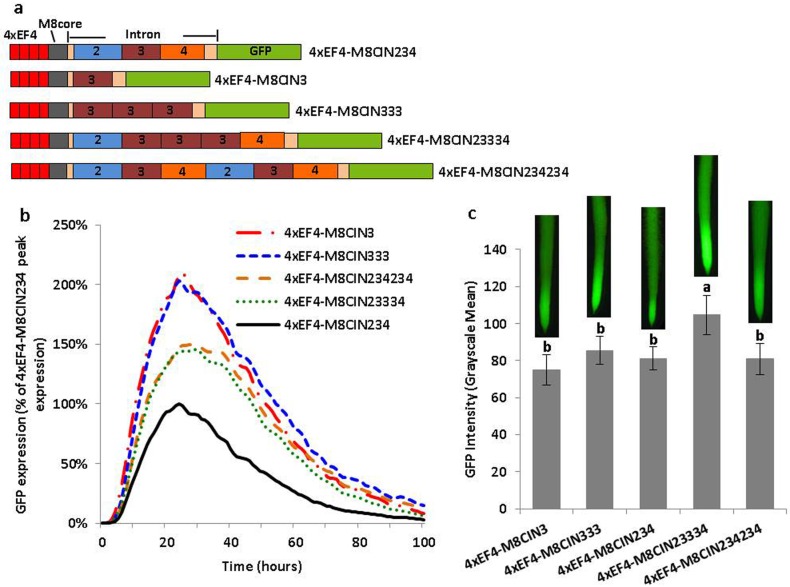
Validation of intronic regulatory sequences using synthetic introns. (a) Schematic of synthetic intron constructs in manipulation of the copy number and sequences of intron regions. (b) Transient GFP expression for various synthetic intron constructs in lima bean cotyledons. The order of constructs in the figure caption key reflects the highest to lowest activity in the curves. (c) GFP intensity value (±SEM) in soybean hairy roots transformed with various synthetic intron constructs. Images of root tips on top of columns represent the average intensity of GFP expression driven by the corresponding promoter construct. Overlapping columns show no significant differences between constructs by the t test (LSD) at p<0.05.

**Fig 7:**
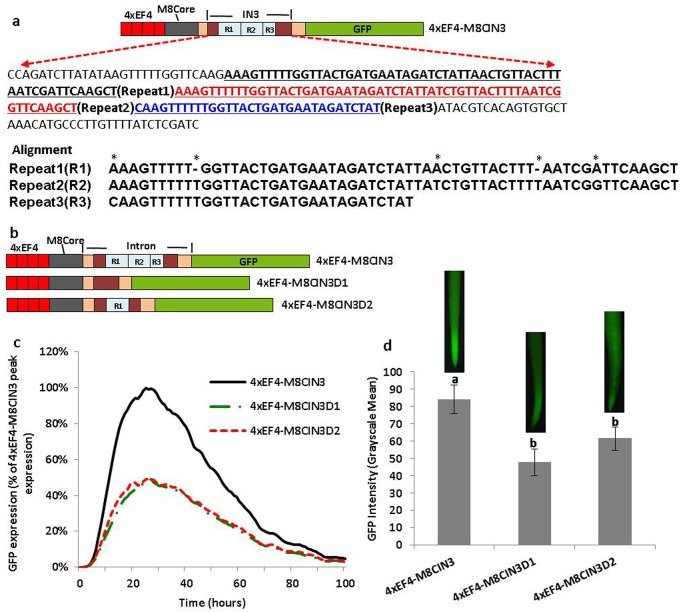
Validation of the repeated sequences in GmScreamM8 leader intron. (a) DNA sequence and alignment of the three imperfect repeated sequences (* indicated mismatches in alignment). (b) Schematic of partial deletion constructs of 4xEF4-M8CIN3. (c) Transient expression profiles for different deletion constructs of the repeated sequences in lima bean cotyledon tissues. The order of constructs in the figure caption key reflects the highest to lowest activity in the curves. (d) GFP intensity value (±SEM) in soybean hairy roots transformed with different deletion constructs of 4xEF4-M8CIN3. Images of root tips on top of columns represent the average intensity of GFP expression driven by the corresponding promoter construct. Overlapping columns show no significant differences between constructs by the t test (LSD) at p<0.05.

Element fragments (EF1, EF4, and EF5; EF: *E**longation* *F**actor 1A*; Figs 3–7 and S1 Fig), containing potential element core sequences predicted by PlantCARE [36] or promoter truncation analysis, were identified from GmScreamM1 (GenBank accession number KX252727) and GmScreamM8 promoters. Element tetramers were generated as described by Rushton et al. [37] with modifications. To generate an element monomer, upper and lower phosphorylated oligonucleotide strands were first annealed together, which created *Spe*I and *Xba*I overhangs in the 5’ and 3’ ends, respectively. The annealed fragment was then introduced into a 35Score-pFLEV plasmid [38], previously digested with *Spe*I and *Xba*I. To double the copy number of elements, each construct was double digested with *Spe*I/*Eco*RI and *Xba*I/*Eco*RI, separately. Ligation of two selected fragments doubled the copy number of element fragments in the plasmid, while retaining all other characteristics of the expression vector [37]. In this way, we generated a series of constructs containing element tetramers, upstream of the 35S core promoter (4xEF1-35Score-pFLEV, 4xEF4-35Score-pFLEV, 4xEF5-35Score-pFLEV [S1 Fig]).

To generate the GmSM8Core-pFLEV construct (100 bp GmScreamM8 core promoter sequence replacing the 46 bp 35S core promoter sequence), a 100 bp region [-60 bp to +40 bp, relative to the transcription start site (TSS)] of GmScreamM8 was initially amplified with GmSM8Core_F/R primers (S1 Table) and then subcloned into pFLEV with appropriate restriction sites. The GmSM8C (GmSM8Core with the native leader intron) in pFLEV was generated as previously described (identical to GmScreamM8C in [34], GenBank accession number KX252740). To generate 4xEF4-M8Core-pFLEV and 4xEF4-M8C-pFLEV constructs (Fig 3), the GmScreamM8 core promoter with or without the leader intron in the GmSM8Core-pFLEV and GmSM8C-pFLEV were digested with *Sal*I/*Nco*I restriction sites and subcloned into 4xEF4-35core-pFLEV to replace the 35S core. To generate 4xEF4-M8CInD series (InD = Intron Deletion; Fig 4), intron partial deletion sequences were PCR-amplified using the GmSM8C_*sal*I_F and GmSM8_R primer pair (S1 Table) with GmSM8InD2-/GmSM8InD3-/GmSM8InD4-pFLEV as templates. The amplified fragments were digested with *Sal*I/*Nco*I and subcloned into the 4xEF4-M8C-pFLEV plasmid digested by the same restriction enzymes.

To generate a series of intron variants with promoter element tetramers (Figs 5–7), inverse PCR was first conducted to add *Avr*II and *Nhe*I sites to the 5’ and 3’ ends of the desired product (4xEF4-M8CInD1-pFLEV or 4xEF4-M8CInD3-pFLEV), respectively. Intron part 2 (IN2), part 3 (IN3), and part 4 (IN4) were PCR-amplified using specific primers with addition of *Avr*II and *Nhe*I restriction sites at the 5’ and 3’ ends, respectively (S1 Table). The copy number of each intronic sequence was manipulated by digesting the constructs with either *Avr*II or *Nhe*I together with *Eco*RI, which cuts the plasmid at a site outside of the synthetic intron region. Ligation of two selected fragments increased the copy number of the intronic sequence. Construct 4xEF4-M8CIN234 (Fig 5a) and 4xEF4-M8C (Fig 4a) both contained tetrameric repeats of EF4 with the GmScreamM8 core promoter and the whole intron but the former construct contained two additional restriction sites (*Avr*II/*Nhe*I) within the intron, which were needed for cloning.

All DNA fragments in this study were PCR-amplified according to the instructions for the FailSafe^‡^ PCR Kit (Epicenter Biotechnologies, Madison, WI, USA). The amplified fragments were purified, digested and ligated to the *gfp* gene in pFLEV, and transformed into *Escherichia coli* DH5α by heat shock. All constructs were confirmed by sequencing. For stable expression in soybean hairy roots, the whole expression cassette containing the promoter, the *gfp* coding region, and the *NOS* terminator was digested from pFLEV with the appropriate restriction enzymes and subcloned into the binary expression vector pCAMBIA1300 (CAMBIA, Canberra, Australia), which was used for *Agrobacterium-*mediated transformation. All pCAMBIA cloned constructs were also confirmed by sequencing.

### Gene Expression Quantification and Data Analysis

For transient expression in lima bean (*Phaseolus lunatus* cv “Henderson Bush”) cotyledonary tissues, lima bean seeds were first sterilized in 4% (v/v) bleach and germinated in GA7 containers containing moistened paper towels. After 4 d, cotyledons from germinating seedlings were excised and used for particle bombardment as described previously [39–40]. In brief, cotyledons were placed, adaxial surface up, on a growth regulator free plant tissue culture medium to acclimate for 1–4 h. Cotyledons were then removed, placed on a stainless steel mesh supporting screen, and bombarded with tungsten particles coated with different DNA constructions using the Particle Inflow Gun [41]. After the cotyledons were returned to the medium, dishes were mounted on a 2 dimensional robotics platform positioned under a MZFLII dissecting fluorescence microscope (Leica, Heerbrugg, Switzerland) and images of each cotyledon were collected every hour for 100 h [40, 42]. To prevent the formation of condensation on the lid of the Petri dish, which would interfere with image capture, the Petri dish lid was replaced with 6.5 mm thick sterile polycarbonate disc [43]. Captured images of cotyledonary tissue showing levels of GFP expression driven by different promoter/element constructs were analyzed as described by Hernandez-Garcia et al. [39]. In brief, each collected series of 100 images for each promoter was first manually aligned using Adobe ImageReady to make certain that the same GFP-expressing area in each cotyledon was analyzed over the 100 h duration of each experimental run. A 400 x 300 pixel area of each image series was selected and used for further analysis of GFP intensity measurement using ImageJ [44]. Batch images were separated into red, green, and blue channels, and background gray values (obtained from a non-GFP expressing region of the cotyledon) were subtracted from each image at each time point. The background-corrected GFP intensity was then calculated by multiplying the mean grayscale value per pixel in the red and green channels by the total number of GFP-expressing pixels to provide a measure of total GFP expression. For each construct, 6–9 cotyledons were bombarded in total, with two or three independent biological replications. The final value of GFP expression for each construct was calculated by averaging the values of GFP expression obtained from the 6–9 individual bombarded cotyledons.

For generation of stably transformed soybean hairy roots, soybean (*G*. *max* cv “Williams 82”) seeds were sterilized and germinated as described above for the lima bean seeds. After 6 d, cotyledons were excised and inoculated with *Agrobacterium rhizogenes* K599 harboring the pCAMBIA1300 vector containing different promoter/element variants [39]. Images of young rapidly growing GFP-expressing hairy roots (~2 cm) were collected using the MZFLIII dissecting microscope equipped with a GFP2 filter set (Ex. 480±40 nm; Em. 510 nm LP), and a Spot-RT CCD digital camera (Diagnostic Instruments Inc., Sterling Heights, MI). GFP intensity was quantified using ImageJ software as previously described [39]. In brief, images of individual roots were separated into red, green and blue channels, and GFP intensity was measured by calculating the background-corrected grayscale mean value using only the green channel. GFP expression values for each promoter construct were calculated by subtracting the grayscale mean value of hairy roots induced by *A*. *rhizogenes* without the binary vector from an average value for the GFP-expressing hairy roots. For each construct, 15–35 independent transgenic events were generated and analyzed, with at least two independent replications. Comparisons between different constructs were analyzed using one-way ANOVA. The significant difference between the means was analyzed using a Student’s t test (LSD) at p<0.05.

### Reverse Transcription PCR (RT-PCR)

Total RNAs of soybean (*G*. *max* cv “Williams 82”) hairy roots (2–3 cm) transformed with or without variant constructs (GmScreamM8, GmSM8ni, GmSM8InD1, GmSM8InD2, GmSM8InD3, and GmSM8InD4) were isolated using the RNeasy Plant Mini Kit (QIAGEN, Valencia, CA, USA). RNA quality was checked by gel electrophoresis and quantified using a Nanodrop spectrophotometer (Thermo Scientific, Wilmington, DE, USA). For reverse transcription PCR (RT-PCR) using a RETROscript^‡^ Kit (AMBION INC, Austin, TX, USA), the first cDNA strand was synthesized using 2 μg RNA and 1 μl random decamers in a reaction volume of 14 μl in total, followed by incubation at 85°C for 3 min, 44°C for 1h, and 92°C for 10 min. Using the synthesized cDNA as template, PCR was performed using specific primers (M8intron_RT_F/R primers; S1 Table) flanking the leader intron. The size of PCR products was checked by gel electrophoresis. PCR products were then gel purified and sequenced to determine if the intron was totally processed and removed.

### IMEter Score

The enhancing ability of the full GmScreamM8 intron or intron fragments was evaluated using IMEter v2.1 (S2 Table) [25]. IMEter scores were calculated by submitting the full GmScreamM8 leader intron or partial intron sequences (part 2, part 3, part 4). “*Glycine max*” (soybean) was selected as the species in the database to evaluate the enhancing ability of the intron/intron fragments.

## Results

This two-tiered validation approach allowed an initial rapid assessment of promoter activity using transient expression, followed by a slightly slower but still rapid analysis in stably-transformed soybean hairy roots. With the constitutively-expressed promoters and regulatory regions selected for this study, gene expression intensities measured using transient expression were largely confirmed in stably-transformed roots.

### The Leader Intron Increased GmScreamM8 (Soybean *eEF1A*) Promoter Activity

To evaluate the effect of the GmScreamM8 leader intron on the high activity of the GmScreamM8 promoter, intron deletion and translocation constructs were generated and validated using both transient expression in lima bean cotyledons and stable expression in soybean hairy roots (Fig 1). The intron-containing GmScreamM8 promoter construct gave much higher transient GFP expression, compared to the intron-less GmSM8ni promoter construct (Fig 1b). Translocation of the intron sequence upstream of the pre-intronic sequence of the GmScreamM8 promoter in both sense (GmSM8InfP) and antisense (GmSM8InrP) orientations yielded lower levels of transient expression compared with the GmScreamM8 promoter, with somewhat higher expression from the upstream placement of the intron in the sense orientation (Fig 1b). Stably-transformed soybean hairy root events from the intron-less (GmSM8ni) and intron translocation constructs (GmSM8InfP; GmSM8InrP) all showed similar expression levels that were not significantly different from each other (Fig 1c). The GmScreamM8 promoter, with the full intron in its native position, gave the highest expression in soybean hairy roots (Fig 1c). The expression patterns were the same for all hairy roots, as all roots displayed higher expression in the root elongation zone and root tips (Fig 1c).

To further dissect the intronic sequences that enhanced gene expression, the full-length native promoter was evaluated with four intron internal deletions (Fig 2). Three of the four intron internal deletion constructs (Fig 2a), GmSM8InD2, GmSM8InD3, and GmSM8InD4, gave rise to a level of transient GFP expression lower than the full intron-containing GmScreamM8 promoter but higher than the intron-less construct GmSM8ni (Fig 2b). In stably transformed soybean hairy roots, the three deletion constructs drove levels of GFP expression significantly lower than the GmScreamM8 promoter but higher than GmSM8ni (Fig 2c). Another large intron deletion construct, GmSM8InD1, in which the majority of the intron sequence (parts 2, 3, and 4) was removed, displayed very weak but detectable activity in both transient and stable expression analyses (Fig 2). Reverse transcription PCR revealed that intron splicing took place with all the constructs except GmSM8InD1, where the intron was not processed, leading to a larger amplicon (Fig 2d, left). This was further confirmed by sequencing.

### The Leader Intron Enhanced the Activity of Proximal Promoter Elements

To simplify the identification of the intronic sequence that contributed to high gene expression, a synthetic promoter consisting of an element tetramer and a core promoter was developed (Fig 3). The element tetramer, EF4, was selected as it showed the highest activity among several element-containing fragments from GmScream promoters (S1 Fig). Different combinations of the EF4 tetramer from GmScreamM1 with the 35S core or GmScreamM8 core promoter, with and without the GmScreamM8 intron, showed that the tetramer with the GmScreamM8 core and GmScreamM8 intron gave the highest activity (Fig 3). This high activity was observed using both transient expression in lima bean cotyledons (Fig 3b) and stable expression in transgenic soybean hairy roots (Fig 3c).

Using transient expression analysis, the construct containing the GmScreamM8 core plus the native leader intron (4xEF4-M8C) showed 33-fold higher expression compared to its counterpart without the intron (4xEF4-M8Core). Another intron-containing construct (4xEF4-35Score-M8Intron) with the 35S core promoter rather than the GmScreamM8 core promoter also displayed significantly enhanced gene expression in comparison with its intron-less counterpart (4xEF4-35Score) (Fig 3b). In addition, the GmScreamM8 core promoter showed seven-fold higher activity when the intron was present (GmSM8C), even if no additional tetrameric promoter sequences were upstream. However, use of the leader intron did not show any enhancement of gene expression when it was cloned downstream of the 35S core promoter without the EF4 tetramer (Fig 3b).

In transformed soybean hairy roots, the 4xEF4-M8C construct showed the highest expression among all the element-intron variants (Fig 3c), similar to what was seen using transient expression analysis. Intron-containing constructs (4xEF4-35Score-M8Intron; 4xEF4-M8C) drove significantly higher GFP expression than the counterparts without the intron (4xEF4-35Score; 4xEF4-M8Core) and the constructs containing only a core promoter and the intron but without the regulatory element tetramer (35Score-M8Intron; GmSM8C) (Fig 3c). For core promoters (35Score or GmSM8Core), the intensity of GFP expression in stably-transformed hairy roots was significantly increased by 2–4 fold if the EF4 tetramer was present upstream (Fig 3c). However, use of either core promoter (35Score or GmSM8Core) without the EF4 tetramer, and either with or without the intron (35Score-M8Intron; GmSM8C), resulted in low but still detectable expression levels (Fig 3c).

### Deletion of a 222 bp Intron Internal Sequence Led to Reduced Activity

Using transient expression analysis of constructs containing the EF4 tetramer, the GmScreamM8 core, and three different intron deletions, reduced expression was obtained only when part 3 of the intron (4xEF4-M8CInD3; Fig 4a) was deleted, while deletion of part 2 (4xEF4-M8CInD2) or part 4 (4xEF4-M8CInD4) did not significantly influence the intensity of gene expression compared to the control 4xEF4-M8C, which possessed a full intron (Fig 4b). In stably transformed hairy roots, GFP expression driven by 4xEF4-M8CInD3 was significantly lower than all other constructs. For the other deletion constructs, 4xEF4-M8CInD2 displayed a significant lower activity than the control 4xEF4-M8C, while 4xEF4-M8CInD4 did not show a significant difference (Fig 4c).

Analysis of three additional intron variants (Fig 5a), where intron parts 2, 3, and 4 were individually evaluated, revealed that the 4xEF4-M8CIN3 gave two-fold higher GFP transient expression than the control 4xEF4-M8CIN234 (same to 4xEF4-M8C with addition of two restriction sites; see Materials and Methods), which possessed the intact intron (Fig 5b). In stably-transformed hairy roots, 4xEF4-M8CIN3 showed comparable activity to 4xEF4-M8CIN234, and significantly higher expression than 4xEF4-M8CIN2 and 4xEF4-M8CIN4 (Fig 5c).

Further analysis of intron variants (Fig 6a) revealed that the monomeric and the trimeric repeat of the 222 bp intronic sequence (4xEF4-M8CIN3; 4xEF4-M8CIN333) gave approximately two-fold higher expression compared to the control 4xEF4-M8CIN234 using transient expression analysis (Fig 6b). A “stuffed” intron (4xEF4-M8CIN23334), which contained a trimeric repeat of intron part 3 and a single copy of intron part 2 and part 4, showed lower expression than the construct containing only one copy of intron part 3 (4xEF4-M8CIN3), but higher expression than the control 4xEF4-M8CIN234. Another intron variant 4xEF4-M8CIN234234, showed 1.5X higher expression than 4xEF4-M8CIN234, but still lower than 4xEF4-M8CIN3 (Fig 6b). For stable expression in soybean hairy roots, the stuffed intron (4xEF4-M8CIN23334) showed the highest expression compared to other intron variants, and a significant difference was observed between the stuffed intron and the control 4xEF4-M8CIN234 (Fig 6c).

DNA sequencing revealed that the 222 bp intronic sequence mainly contained an array of two and a half short imperfect repeats, in lengths of 60 bp, 60 bp, and 30 bp (Fig 7a and 7b). Deletion of the whole repeated sequence within the intron (4xEF4-M8CIN3D1) led to 50% decrease of GFP expression in transient expression as well as a significant decrease in GFP expression in stably-transformed hairy roots, while retention of the first 60 bp repeat sequence (4xEF4-M8CIN3D2) did not restore the activity of the whole repeated sequence (4xEF4-M8CIN3), as evaluated using both transient and stable expression (Fig 7c and 7d).

## Discussion

### Transient Expression

Use of the automated image collection and analysis system with GFP allowed for continual monitoring of promoter activity over 100 h (Figs 1–7), generating consistent results for measurement of promoter strength. This approach has been used previously with constitutively expressed promoters [26, 39] as well as wound-inducible promoters [39] and promoter elements [38]. This same transient expression approach was also used for rapid validation of the GmScream promoters [34], and showed that the GmScreamM1, GmScreamM4 and GmScreamM8 promoters were the most highly active promoters in this group. The specific *gfp* gene that was used in this research is an earlier version of the gene and the protein displays moderate fluorescence and stability [45], compared with other green fluorescent proteins, which have been modified for increased fluorescence and higher stability [46–47]. The GFP fluorescence reported here should therefore be more indicative of promoter activity than other reporter genes, which give rise to more stable products and may still be measured, long after the promoter becomes inactive. Although it may have been useful to evaluate either GUS or luciferase activity, regulated by a constitutive promoter, as an internal standard [48], this approach was not feasible using a continual monitoring system as GUS and luciferase assays are destructive and can only be used for single time point assays. In this research, GFP expression was evaluated and quantified over time based on image analysis of a defined area of GFP-expressing cells in bombarded tissue. GFP levels were calculated based on background-corrected average gray value in the green and red channels across thousands of transiently expressing cells [42], in 6–9 cotyledons, from 2–3 separate experiments. This approach has been useful for quantification of the effects of promoters [39], intron-variants [26] and *cis*-regulatory elements [38], and seems to be useful for evaluation of the promoter and intron variants described in this research.

### Stable Expression in Hairy Roots

Promoter-mediated gene expression levels in stably transformed soybean hairy roots were largely reflective of the levels obtained using transient expression analysis (Figs 1–5 and 7), with slight inconsistency across systems in certain instances (Fig 6). Use of hairy roots to validate promoter constructs may lead to slightly different outcomes, as transgene expression in hairy roots and other transgenic organs is influenced by genome methylation as well as conformational and positional effects in genomic DNA [39]. GFP expression in stably transformed roots showed some variability, which was previously demonstrated to be associated with transgene copy number [39]. Our rapid and simple stable transformation assays in soybean hairy roots was extremely useful for rapid introduction and validation of many different promoter constructs, since recovery of transgenic soybean plants requires extended time and effort [49]. In this study, production of 15–35 stably transformed hairy roots for each promoter construct, from two independent experiments, took weeks rather than months. In addition, hairy roots are well suited for GFP detection, as they do not contain chlorophyll, which can interfere GFP detection due to chlorophyll autofluorescence [50]. Although use of hairy roots for promoter analysis does have some limitations, evaluation of gene expression in stably transformed soybean hairy roots can be a good general indicator of promoter strength in whole transgenic plants [26, 34, 38].

### Intron-Mediated Enhancement

Removal of the leader intron from the original GmScreamM8 promoter (GmSM8ni, Fig 1) resulted in 60% decrease in gene expression, suggesting that the leader intron significantly contributed to the high activity of the ~1.5 kb promoter (Fig 1). The GmScreamM8 leader intron is located only 77 bp downstream of the TSS of the native soybean *eEF1A* gene. As with other stimulating introns, the close proximity of the 5’UTR intron to the TSS may allow better access of the transcriptional initiation machinery to the intron [51–52], enhancing transcription initiation. In addition, the first introns relative to other introns of the same gene usually display significant enrichment of active chromatin marks, further indicating certain regions in first introns have potential regulatory roles [53].

Introduction of intron translocation constructs (GmSM8InfP and GmSM8InrP) (Fig 1) did not lead to high GFP expression, suggesting that the GmScreamM8 leader intron did not contain classical enhancers that could increase gene expression independent of the position and orientation. Using the same validation tools reported here, upstream translocation of the leader intron from the soybean *polyubiquitin* (Gmubi) promoter in both orientations led to increased expression levels, indicating that enhancers were present in that intron sequence [26]. Unlike the Gmubi intron, the GmScreamM8 leader intron enhanced gene expression via intron-mediated enhancement, as the stimulating intron needed to be placed close to, and downstream of the transcription start site in the proper orientation [54].

Partial deletion of intronic regions (part 2, part 3, part 4) in GmScreamM8 did not completely abolish intron enhancement of gene expression (Fig 2), but resulted in different levels of reduction in expression, indicating a redundancy of IME signals, which is common with this type of intron [55], possibly due to the interactions of intronic regulatory sequences throughout the GmScreamM8 intron and the multiple proximal promoter regulatory motifs. Redundancy of IME signals can complicate the identification of intron sequence motifs responsible for intron enhancement, because each motif can influence IME in different ways, depending on the number of intron regulatory motifs and the proximity of the motifs to the promoter elements.

Analysis of intron partial deletion constructs with the full-length promoter showed that the partial deletions reduced promoter activity (Fig 2). The largest intron deletion construct GmSM8InD1 (Fig 2), with only a 68 bp intron sequence remaining, showed very low but detectable GFP expression in both transient and stable expression assays. This was the only intron-containing construct, where the intron was not processed (Fig 2d). The failure in intron processing with the GmSM8InD1 construct likely affected mRNA stability and translation efficiency by interruption of the reading frame [54], or by triggering nonsense-mediated mRNA decay (NMD) [56]. Splicing failure of GmSM8InD1 could have resulted from a short intron size [57], where removal of the majority of the intronic regulatory elements/enhancers resulted in very inefficient splicing. However, the role of splicing on IME is still not clear. Splicing alone is not sufficient to induce IME [58] since not all spliceosomal introns can enhance gene expression [25]. But, splicing is often necessary for IME [32].

### Use of Synthetic Promoters and Synthetic Introns for Intron Sequence Evaluation

Use of a full-length promoter upstream of a stimulating intron clearly demonstrated an interaction between the promoter and the GmScreamM8 leader intron (Figs 1 and 2) but it was difficult to identify the interacting components using the full-length promoter and the intron. Although IME has previously been studied using full-length native promoters along with introns and intron deletion variants [22, 31, 59–60], use of synthetic promoters consisting of an element tetramer and a minimal promoter to study IME has not previously been reported.

Although the 35S core has been widely used for evaluation of *cis*-regulatory elements due to its low background expression and efficient transcriptional initiation [61], the GmScreamM8 core was more efficient than the 35S core for intron sequence evaluation, as the leader intron exhibited a larger gene enhancement with the GmScreamM8 core compared to the 35S core using our transient and stable expression assays, with or without the element tetramer upstream of core promoters (Fig 3). Use of our synthetic promoters, consisting of the EF4 tetramer and the GmScreamM8 core promoter assembled upstream of the intact intron or intron variants (Figs 4–7), allowed for a robust assessment of the interaction between the promoter element and the intron components, with no apparent interference from additional regulatory sequences in the full-length promoter. Our modified plasmids facilitated the evaluation of candidate intronic sequences with small tetrameric promoter fragments, using rapid transient expression analysis and stable expression assays.

Use of synthetic introns (Figs 6 and 7) holds great potential for identification and evaluation of intron regulatory elements conferring IME. With synthetic introns, intron sequences of interest can be evaluated by manipulating the copy number and sequences of intron regions (Fig 6). These types of intron variants, in addition to the intron translocation constructs (Fig 1) and the partial internal deletion constructs (Figs 4, 5 and 7), can be very useful for identifying intron components that contribute to increased gene expression. As demonstrated here, evaluation of synthetic intron constructs using synthetic promoters, consisting of various core sequences with tetrameric promoter element repeats has not previously been reported.

### Intron Regulatory Motifs for IME

Our results suggested that a 222 bp intronic sequence (part 3) was an important contributor to IME from the GmScreamM8 leader intron (Figs 4–6). Further truncation of the repeated sequence within the intron led to dramatic reductions in GFP expression (Fig 7), indicating that the whole repeated sequence contributed to high levels of gene expression. The repeated sequence may contribute to conformational changes, making the promoter more accessible for transcription factors and RNA polymerase II [62], or facilitating the interaction of splicing factors with upstream transcription factors [27]. Alternatively, the repeated sequence possibly contained regulatory sequences involved in IME; however, we were not able to identify any candidate intron regulatory sequences using ERISdb [63], possibly due to the limited knowledge of intron regulatory elements in plants. Nevertheless, we found that the repeated sequence was T-rich, with T-nucleotides accounting for more than 40% of the whole sequence. A T-rich region within the maize *Sh1* first intron was required for the maximum levels of intron enhancement [32]. In addition, a TAGATC was found in the three imperfect repeated sequences of our GmScreamM8 leader intron (Fig 7). The TAGATC sequence was similar to an overrepresented sequence of TA/CGATC/G in rice stimulating introns [64] and a pentamer CGATT in the Arabidopsis *UBQ10* intron [25, 64], which were identified as important regulatory elements for IME. For the other two intron fragments studied here, the part 2 region showed very limited effects on the IME, while the part 4 displayed a negative effect (Figs 4–6), indicating silencers or regulatory sequences inhibiting gene transcription existed in the part 4 of the intron. Analysis of the IMEter scores of the different intron parts using IMEter v2.1 [25] revealed that the intron part 2 region had the strongest IME signal (S2 Table). Although IME signals may show conserved features among some plants [25], analysis of the leader intron using IMEter v2.1 did not reveal the sequence that contributed to high gene expression.

Taken together, the middle part of the GmScreamM8 leader intron (part 3), which contained potential regulatory elements for IME, seemed to contribute greatly to the enhancement of GFP expression, while the part 4 of the intron may contain negative regulatory sequences for the regulation of gene expression.

## Conclusions

We report here an interplay between a leader intron from a native soybean GmScreamM8 promoter and a regulatory element of the GmScreamM1 promoter to regulate gene expression. The GmScreamM8 leader intron increased gene expression through intron-mediated enhancement. Using a synthetic promoter approach and synthetic introns, we identified a short repeated intronic region of the GmScreamM8 leader intron that significantly contributed to the observed IME. This approach could be useful for the identification and validation of other intron regulatory components for gene enhancement, thus improving our understanding of the mechanisms of IME. The GmScreamM8 intron and possibly other introns have the potential to increase, stabilize or modulate transgene expression when used in transgenic plants, either in their native form, or as synthetic introns.

## Supporting Information

S1 Fig Validation of synthetic promoters containing tetrameric copies of element-containing fragments. (a) DNA sequences of EF1, EF4 and EF5. Underlines are potential elements within the fragments. (b—e) Profiles of transient GFP expression in lima bean cotyledons driven by different synthetic promoters containing either the 35S core promoter or the GmScreamM8 core promoter with or without the GmScreamM8 leader intron.
(DOCX)

S1 File DNA sequences for the GmScreamM8 intron and intron fragments. (DOCX)

S1 Table List of primer sequences used for PCR amplification. (DOCX)

S2 Table IMEter scores for the GmScreamM8 intron and intron fragments. (DOCX)
