# Supplementary material for: A Leader Intron of a Soybean Elongation Factor 1A (eEF1A) Gene Interacts with Proximal Promoter Elements to Regulate Gene Expression in Synthetic Promoters
Source: PLoS One. 2016 Nov 2;11(11):e0166074. doi: 10.1371/journal.pone.0166074 (PMC5091777; doi:10.1371/journal.pone.0166074)

**S1 Fig. Validation of synthetic promoters consisting of tetrameric copies of element-containing fragments.** (a) DNA sequences of EF1, EF4 and EF5. Underlines are potential elements within the fragments. (b-e) Profiles of transient GFP expression in lima bean cotyledons driven by different synthetic promoters containing either the 35S core promoter or the GmScreamM8 core promoter with or without the GmScreamM8 leader intron.


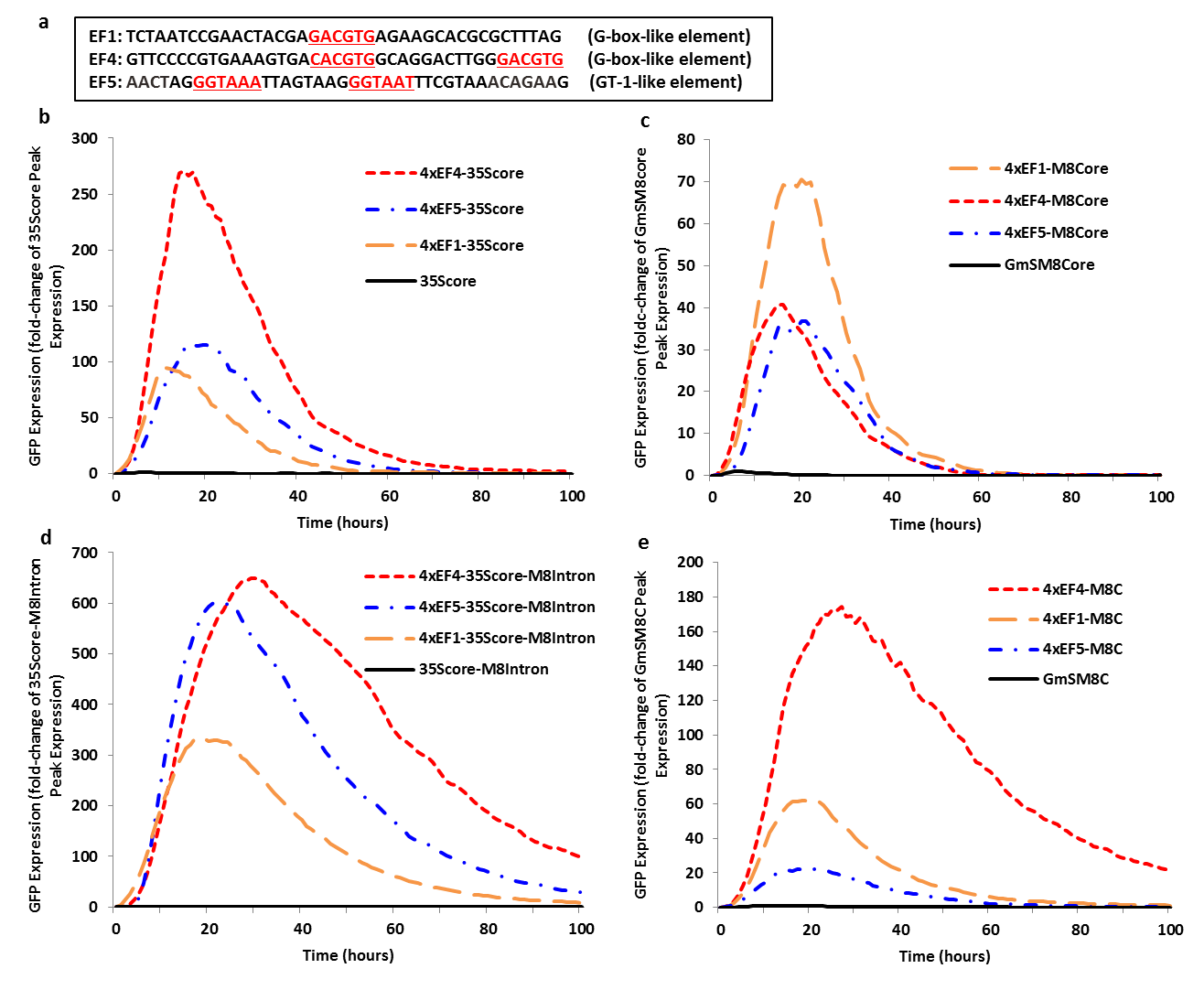

Supplement: S1 Fig — (a) DNA sequences of EF1, EF4 and EF5. Underlines are potential elements within the fragments. (b—e) Profiles of transient GFP expression in lima bean cotyledons driven by different synthetic promoters containing either the 35S core promoter or the GmScreamM8 core promoter with or without the GmScreamM8 leader intron. (DOCX) [file pone.0166074.s001.docx]
