# Supplementary material for: A Leader Intron of a Soybean Elongation Factor 1A (eEF1A) Gene Interacts with Proximal Promoter Elements to Regulate Gene Expression in Synthetic Promoters
Source: PLoS One. 2016 Nov 2;11(11):e0166074. doi: 10.1371/journal.pone.0166074 (PMC5091777; doi:10.1371/journal.pone.0166074)
Supplement: S1 File — (DOCX) [file pone.0166074.s002.docx]

**S1 File. DNA sequences for the GmScreamM8 intron and intron fragments.**

>Full intron

**GT**TCGTTATCTACCACCGTTCTATGGATTTTATTCCTTCTATTCGTGTTTATTCTATTGGTTTATGTTGCTTGCAATATGTTTTTTCTGAATCTGTCGTCGTTGTCTTCAATTTTATCCATGTTTCAGAGATCAATTTTGTTTGTGTAGTATGTGCTTATTCTTCTTCTTTTCGTTCGAGTTGTTAATAACGGTGCTATGGTGTTTTCAAAAGTGTTTTTTTTATTACTTTTGATTTAAAGTTTTTTTGGTAAGGCTTTTATTTGCTTGTTATATTCAAATCTTTGGATCCAGATCTTATATAAGTTTTTGGTTCAAGAAAGTTTTTGGTTACTGATGAATAGATCTATTAACTGTTACTTTAATCGATTCAAGCTAAAGTTTTTTGGTTACTGATGAATAGATCTATTATCTGTTACTTTTAATCGGTTCAAGCTCAAGTTTTTTGGTTACTGATGAATAGATCTATATACGTCACAGTGTGCTAAACATGCCCTTGTTTTATCTCGATCTTATGTATGGGAGTGCCATAAATTTTGTTATGTCTATTTTTTTATCTGTTGGAATCATACTGAGTTTGATGCGTTACGATTGAGCATACCTATTTTTGGGCTTGTTGTATGGTGGGTATTTAGATCTTAATCTTTTTATGCTTATGAAAGGTTTTGTAATGACAAAGGTCTTAATGTTGTTAAACTTTTATTTTTACTTTATATGGTGTGTTGATGTGTTATGGTTTTGACAACTTTTTTTTTTTCTGGATTTTTGC**AG** (**GT**: donor splice site; **AG:** acceptor splice site; TTTTGAC is branch site, predicted by SVM-BPfinder: http://regulatorygenomics.upf.edu/Software/SVM_BP/)

>Intron part2

CTATGGATTTTATTCCTTCTATTCGTGTTTATTCTATTGGTTTATGTTGCTTGCAATATGTTTTTTCTGAATCTGTCGTCGTTGTCTTCAATTTTATCCATGTTTCAGAGATCAATTTTGTTTGTGTAGTATGTGCTTATTCTTCTTCTTTTCGTTCGAGTTGTTAATAACGGTGCTATGGTGTTTTCAAAAGTGTTTTTTTTATTACTTTTGATTTAAAGTTTTTTTGGTAAGGCTTTTATTTGCTTGTTATATTCAAATCTTTGGAT

>Intron part3

CCAGATCTTATATAAGTTTTTGGTTCAAGAAAGTTTTTGGTTACTGATGAATAGATCTATTAACTGTTACTTTAATCGATTCAAGCTAAAGTTTTTTGGTTACTGATGAATAGATCTATTATCTGTTACTTTTAATCGGTTCAAGCTCAAGTTTTTTGGTTACTGATGAATAGATCTATATACGTCACAGTGTGCTAAACATGCCCTTGTTTTATCTCGATC

>Intron part4

TTATGTATGGGAGTGCCATAAATTTTGTTATGTCTATTTTTTTATCTGTTGGAATCATACTGAGTTTGATGCGTTACGATTGAGCATACCTATTTTTGGGCTTGTTGTATGGTGGGTATTTAGATCTTAATCTTTTTATGCTTATGAAAGGTTTTGTAATGACAAAGGTCTTAATGTTGTTAAACTTTTATTTTTACTTTATATGGTGTGT
