# Supplementary material for: A Leader Intron of a Soybean Elongation Factor 1A (eEF1A) Gene Interacts with Proximal Promoter Elements to Regulate Gene Expression in Synthetic Promoters
Source: PLoS One. 2016 Nov 2;11(11):e0166074. doi: 10.1371/journal.pone.0166074 (PMC5091777; doi:10.1371/journal.pone.0166074)
Supplement: S1 Table — (DOCX) [file pone.0166074.s003.docx]

**S1 Table. List of primer sequences used for PCR amplification.**

| **Promoter** | **Primer sequence (5’ to 3’)** | |
| --- | --- | --- |
| **GmSM8ni** | F: GCGCGCAAGCTTTTACGTGTGCTGTGAGACATTATC (*Hin*dIII) | |
|  | R:GAGACCATGGTCCTTAAATCTAGAAGAAGCTGCGCTAAAACC (*Nco*I) | |
| **GmSM8Inf(r)P** | F: GAGAAAGCTTTCTAGGTTCGTTATCTACCA (*Hin*dIII) | |
|  | R:GAGAAAGCTTCTTAAATCTGCAAAAATCCAGA (*Hin*dIII) | |
| **GmSM8INDel1** | F:TGATGTGTTATGGTTTTGACAAC | |
|  | R:AACGGTGGTAGATAACGAAC | |
| **GmSM8INDel2** | F:CCAGATCTTATATAAGTTTTTGGTTC | |
|  | R:AACGGTGGTAGATAACGAACCTA | |
| **GmSM8INDel3** | F:CATGCCCTTGTTTTATCTCGATC | |
|  | R:GAACCAAAAACTTATATAAGATCTGG | |
| **GmSM8INDel4** | F:TGATGTGTTATGGTTTTGACAAC | |
|  | R:GATCGAGATAAAACAAGGGCATG | |
| **GmSM8C** | F: GAGCAAGCTTTTCGTAAACAGAAGAAAAGAGTTG (*Hin*dIII) |  |
| **GmSM8C*-sal*I** | F: GAGCGAGTCGACTTCGTAAACAGAAGAAAAGAGTTG (*Sal*I) | |
| **GmSM8Core** | F: GAGCAAGCTTTTCGTAAACAGAAGAAAAGAGTTG (*Hin*dIII) | |
|  | R: GCGACCATGGGCCGCAAGAGAAGAAGAGTG (*Nco*I) | |
| **GmSM8Core*-sal*I** | F: GAGCGTCGACTTCGTAAACAGAAGAAAAGAGTTG (*Sal*I) | |
| **GmSM8** | R: TGCGCGCCATGGTCTTCGCTCTTCACTCTTGCTC (*Nco*I) | |
| **EF1** | F:CTAGTTCTAATCCGAACTACGAGACGTGAGAAGCACGCGCTTTAGT | |
|  | R:CTAGACTAAAGCGCGTGCTTCTCACGTCTCGTAGTTCGGATTAGAA | |
| **EF4** | F:CTAGTGTTCCCCGTGAAAGTGACACGTGGCAGGACTTGGGACGTGT | |
|  | R:CTAGACACGTCCCAAGTCCTGCCACGTGTCACTTTCACGGGGAACA | |
| **EF5** | F:CTAGTAACTAGGGTAAATTAGTAAGGGTAATTTCGTAAACAGAAGT | |
|  | R:CTAGACTTCTGTTTACGAAATTACCCTTACTAATTTACCCTAGTTA | |
| **M8CINDel1_*Nhe*I** | F:GAGCGACCTAGGTGATGTGTTATGGTTTTGACAAC (*Avr*II) | |
|  | R:GGATCCGCTAGCAACGGTGGTAGATAACGAAC (*Nhe*I) | |
| **M8CINDel3_*Nhe*I** | F:GAGCGACCTAGGTTATGTATGGGAGTGCCATAAATTTTG (*Avr*II) | |
|  | R:GGATCCGCTAGCATCCAAAGATTTGAATATAACAAGCAAA (*Nhe*I) | |
| **M8IN2** | F:GAGCGAGCTAGCCTATGGATTTTATTCCTTCTATTC (*Nhe*I) | |
|  | R:GAGCGACCTAGGATCCAAAGATTTGAATATAACAAGC (*Avr*II) | |
| **M8IN3** | F:GAGCGAGCTAGCCCAGATCTTATATAAGTTTTTGGTTC (*Nhe*I) | |
|  | R:GAGCGACCTAGGGATCGAGATAAAACAAGGGCATG (*Avr*II) | |
| **M8IN4** | F:GAGCGAGCTAGCTTATGTATGGGAGTGCCATA (*Nhe*I) | |
|  | R:GAGCGACCTAGGACACACCATATAAAGTAAAAA (*Avr*II) | |
| **M8IN234** | F:GAGCGAGCTAGCCTATGGATTTTATTCCTTCTATTCGTG (*Nhe*I) | |
|  | R:GAGCGACCTAGGACACACCATATAAAGTAAAAATAAAG (*Avr*II) | |
| **M8CIN3Del1** | F:ATACGTCACAGTGTGCTAAACATG | |
|  | R:CTTGAACCAAAAACTTATATAAGATCTG | |
| **M8CIN3Del2** | F:ATACGTCACAGTGTGCTAAACATG | |
|  | R:TAGCTTGAATCGATTAAAGTAACAGTTAAT | |
| **M8Intron_RT** | F:GTTCACTCTTCTTCTCTTGCGGCTA | |
|  | R:CGTAGGTGAAGGTGGTCACGAG | |

F-forward primer, R-reverse primer. Restriction sites are underlined.
