# Supplementary material for: A Leader Intron of a Soybean Elongation Factor 1A (eEF1A) Gene Interacts with Proximal Promoter Elements to Regulate Gene Expression in Synthetic Promoters
Source: PLoS One. 2016 Nov 2;11(11):e0166074. doi: 10.1371/journal.pone.0166074 (PMC5091777; doi:10.1371/journal.pone.0166074)
Supplement: S2 Table — (DOCX) [file pone.0166074.s004.docx]

**S2 Table. IMEter scores for the GmScreamM8 intron and intron fragments.**

| **Intron/intron fragments** | **Length (bp)** | **IMEter score** | **Percentile*** |
| --- | --- | --- | --- |
| **Full intron** | 770 | 16.03 | 98 |
| **Intron part2** | 269 | 11.47 | 96 |
| **Intron Part3** | 222 | 4.61 | 77 |
| **Intron Part4** | 211 | 3.54 | 68 |

*The percentile of the score for all introns of soybean genes
